# Central Nervous System Aspergillosis: An Unexpected Complication following Neurosurgery

**DOI:** 10.3390/diseases6020046

**Published:** 2018-05-31

**Authors:** Jose Armando Gonzales Zamora, Zachary Henry, Sakir Humayun Gultekin

**Affiliations:** 1 Division of Infectious Diseases, Department of Medicine, Miller School of Medicine, University of Miami, 1120 NW 14th Street, Suite 863B, Miami, FL 33136, USA; zachary.henry@jhsmiami.org; 2 Division of Anatomic Pathology, Department of Pathology and Laboratory Medicine, Miller School of Medicine, University of Miami, Miami, FL 33136, USA; sgultekin@miami.edu

**Keywords:** post-surgical, central nervous system, *Aspergillus*, PCR, voriconazole

## Abstract

Post-surgical aspergillosis is an uncommon complication that carries a high mortality rate in affected patients. The diagnosis is challenging given the lack of highly sensitive methods to isolate *Aspergillus* from surgical sites. Here, we present a case of post-surgical aspergillosis that occurred after the resection of acoustic neuroma in an immunocompetent patient. Imaging revealed leptomeningeal enhancement and a cerebellar extra-axial fluid collection adjacent to the right retrosigmoid craniotomy. The patient was taken to the operating room for debridement, where purulent fluid was obtained from subdural space. The diagnosis was achieved by histopathology and polymerase chain reaction (PCR) in brain tissue. Appropriate investigations failed to detect contamination in the operating room. The patient was successfully treated with 3 months of voriconazole. We highlight the importance of recognizing this uncommon complication and advocate for the use of molecular techniques to improve the diagnostic yield in central nervous system aspergillosis.

## 1. Introduction

Aspergillosis is an infection caused by fungi of the genus *Aspergillus*. The most common human pathogen of this species is *Aspergillus fumigatus*, but *A. flavus* and *A. niger* are also frequently reported. *Aspergillus* is a ubiquitous fungus, and its normal ecological niche is the soil, water, and decaying vegetation. It has been recognized as a major cause of disease in immunocompromised individuals, especially in the setting of intensive chemotherapy, hematopoietic stem cell transplantation, or solid organ transplantation. Post-operative aspergillosis is a rare complication after surgical procedures and occurs mainly in immunocompetent patients whose predisposing condition is a breach of the skin or mucosal barriers. It has been described after many surgical interventions, and dental and ophthalmologic surgery are the most common procedures to be associated with this complication [1]. We report a case of post-surgical subdural empyema in a patient who had recent craniotomy for acoustic neuroma resection, and who was treated successfully with medical and surgical therapy. In this report, we highlight the importance of recognizing this uncommon pathology in patients with recent surgical interventions, and discuss the challenges we face for the diagnosis and treatment of postsurgical aspergillosis.

## 2. Case Description

**Figure 1:**
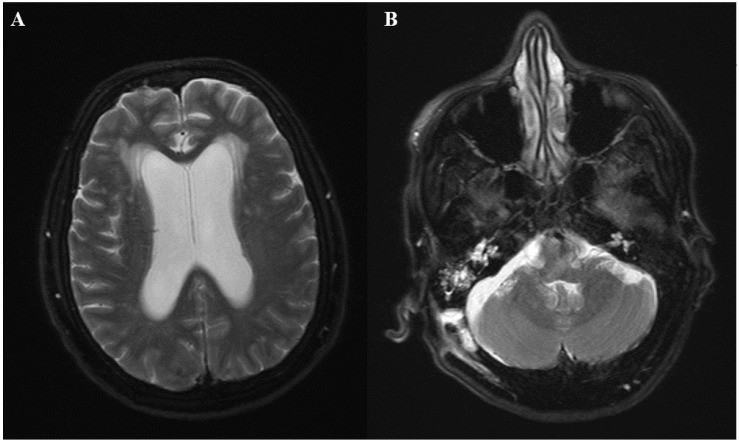
(**A**) Axial T2-weighted MRI (magnetic resonance imaging)showing diffuse leptomeningeal enhancement and moderately dilated ventricles (**B**) T2-weighted MRI showing a right cerebellar extra-axial collection of approximately 1 cm in diameter.

**Figure 2:**
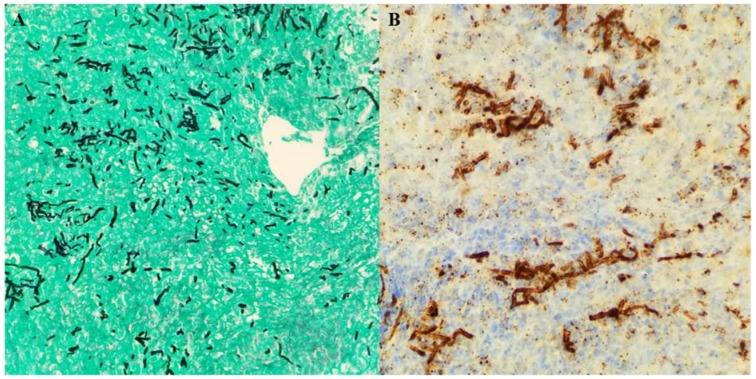
(**A**) Histopathology showing fungal elements in hyphal form with acute branching and vague septae (Gomori Methenamine Silver stain, 100×) (**B**) Positive immunohistochemistry for *Aspergillus* (Immunostaining, 200×).

A 71 year-old woman with a history of hypertension, hypothyroidism, and acoustic neuroma was admitted for right retrosigmoid craniotomy and tumor resection. Her operation was uneventful, with no immediate post-surgical complications. She was discharged on dexamethasone 6 mg daily for 3 days, with tapering doses of steroids over the course of 1 week. Three weeks later, she presented to the hospital with persistent fever and chills, and had experienced occasional headaches for five days. On admission, her vitals were significant with a temperature of 38.5 °C, heart rate of 83 beats per minute, respiratory rate of 14 breaths per minute, and blood pressure of 158/83 mmHg. On examination, she was alert and oriented to person, place, and time. Her right posterior auricular incision site looked clean, with mild erythema but no discharge. Sutures were intact. Neurologic exam did not reveal any focal or meningeal signs. Laboratory studies were significant with a white blood cell count of 8.9 k/μL, hemoglobin of 10.9 g/dL, and a platelet count of 313 k/μL. Urinalysis showed 33 white blood cells, positive leukocyte esterase, and negative nitrates. Urine culture grew more than 100,000 colonies of *Klebsiella pneumoniae.* She was started on cefepime 2 g intravenously every 12 h for presumptive urinary tract infection. Despite antibiotic coverage for 3 days, the patient continued to have fevers up to 38.7 °C. Given her recent surgical intervention, a computed tomography (CT) scan was ordered, which showed a subgaleal collection overlying the craniotomy site and hypodense areas in the right cerebellum that likely represented postoperative changes. A hypodense extra-axial collection was noted along the right cerebellum. Given the concern for postsurgical meningitis, cefepime was switched to meropenem 2 g every 8 h, and vancomycin 1 g every 12 h was added to her antibiotic regimen. The patient showed a rapid clinical deterioration, with the development of nuchal rigidity, altered mental status, and seizures in the next 24 h. She underwent a lumbar puncture, which evidenced an opening pressure of 30 cm H_2_O. Cerebrospinal fluid (CSF) analysis showed 800 white blood cells/mm^3^ with 90% neutrophils, low glucose (31 mg/dL), and high protein (115 mg/dL). The smear for acid-fast bacilli in CSF was negative. CSF Cryptococcus antigen was also negative. The FilmArray^®^ meningitis/encephalitis panel did not identify any organisms. Emergent magnetic resonance imaging (MRI) was ordered, which showed diffuse leptomeningeal enhancement and moderately dilated ventricles. Fluid layering within the bilateral occipital horns was noted, as there were concerns of ventriculitis. Additionally, a 1 cm right cerebellar extra-axial collection was seen (Figure 1A,B). The patient was immediately taken to the operating room for the placement of an external ventricular drain. Intrathecal colistin and vancomycin were initiated afterwards. Follow-up CSF analysis 48 h later showed normalization of white blood cell count (2 cells/mm^3^); however, low glucose and high protein persisted. Lactic acid from CSF was elevated (35 mg/dL). Bacterial and fungal cultures from the blood and CSF did not show any growth. Intraventricular antibiotics were discontinued after 48 h, and the patient continued with meropenem and vancomycin intravenously for 2 weeks. Despite antibiotic therapy, the patient’s mental status did not improve significantly, and the fevers continued intermittently. Further studies that included serum galactomannan and (1→3)-β-d-glucan were negative. A repeat MRI showed persistent leptomeningeal enhancement at the cervicomedullary junction at the skull. Extra-axial collection adjacent to the retrosigmoid craniotomy was again noted. A CT scan of the chest, abdomen, and pelvis was ordered to rule out other potential sources of infection, but imaging was unremarkable. On further questioning, the patient’s daughter stated that her mother was in India and Nepal three months prior to the initial craniotomy, where she was working as a volunteer in a clinic and was exposed to patients with tuberculosis (TB). The patient had a history of a positive purified protein derivative (PPD), but was never treated for latent TB. Given her epidemiologic risk factors, MRI findings and clinical deterioration, the patient was started on isoniazid, rifampin, pyrazinamide, and levofloxacin for the treatment of presumptive tubercular meningitis. However, after one week of treatment, no clinical improvement was noted, and she was taken to the operating room for exploration. The patient underwent midline limited suboccipital craniotomy and C1 laminectomy. Clear pus was noted in the area of the foramen magnum, foramina of Magendie, and posterior to the upper cervical cord, with evidence of pachymeningitis of the pia of the upper cervical cord and both cerebellar tonsils. The subdural empyema was evacuated and samples were sent for cultures. A biopsy of the arachnoid tissue was obtained. Intra-operative frozen section revealed chronic mixed inflammatory, but no granulomas. Two days later, histopathology reported fungal elements. Immunohistochemistry for *Aspergillus* was strongly positive (Figure 2A,B). Toxoplasma, acid-fast bacilli, and gram stains were negative. Tissue sample was sent to the University of Washington in Seattle, WA for fungal PCR. Anti-tuberculosis treatment was discontinued and the patient was started on liposomal amphotericin 5 mg/kg daily and voriconazole intravenously 6 mg/kg every 12 h as a loading dose, and then 4 mg/kg every 12 h for maintenance. Five days later, tissue real-time PCR came back positive for *Aspergillus fumigatus.* Amphotericin was discontinued and the patient was kept on voriconazole. The patient’s mental status improved markedly over the following days. The fever subsided as well. Treatment with intravenous voriconazole was eventually transitioned to oral voriconazole 250 mg every 12 h to complete 3 months of therapy. The voriconazole level at 2 weeks of treatment was 2.6 μg/mL, which was within the therapeutic range for central nervous system (CNS) aspergillosis (reference range 2–5 μg/mL). The patient was later transferred to a rehabilitation facility for physical therapy. At the four month follow-up, the patient was fully alert, and her verbal communication was almost back to her baseline. No focal or meningeal signs were noted on examination. A brain MRI was repeated and showed complete resolution of leptomeningeal enhancement, with no collections.

## 3. Discussion

Post-surgical aspergillosis is an uncommon complication, affecting 2 in every 10,000 surgical procedures [2]. It is caused by an external source of *Aspergillus* conidia contaminating normally sterile sites during or after surgery. Many types of surgical procedures have been linked to this complication such as thoracic, cardiac, vascular, neurological, and dental surgical interventions. According to Pasqualotto et al., the procedures with the highest risk of post-surgical aspergillosis are cardiac, dental, and ophthalmologic surgeries [1]. The retrospective review of the literature conducted by these authors in 2015 identified 25 cases related to neurosurgery [1]. In many cases, the exact pathway by which *Aspergillus* reached the brain could not be determined. In immunocompromised individuals, the pathophysiology of cerebral aspergillosis is different, and it may occur by direct extension from the ear, nose, or paranasal sinuses, or after hematogenous dissemination from a primary focus in the lung.

Many studies have associated post-surgical aspergillosis with dissemination of *Aspergillus* conidia in the operating room. An investigation conducted by Heinemann et al. after an outbreak of sternal surgical site infection found that massive contamination by *A. flavus* occurred in some areas of the surgical ward [3]. By surface sampling, they were able to localize a high gradient of contamination in these zones. Furthermore, molecular typing demonstrated the same genotype for the species isolated, which proved the clonal single-source of the environmental contamination and the intra-operative acquisition of *Aspergillus flavus* [3]. Others have reported cases of nosocomial aspergillosis linked to a break in the high efficiency particulate air (HEPA) filtration system [4]. In our patient’s circumstance, appropriate investigations in the operating room were performed. Air samples were collected using the Merck air sampler (Millipore Sigma, Burlington, MA, USA) and were then cultured on Sabouraud-chloramphenicol agar plates. No *Aspergillus* colonies were observed. We also analyzed samples from purified water and did not identify any mold species.

Another interesting phenomenon is the occurrence of aspergillosis outbreaks following natural disasters. After the tsunami of 2005, an outbreak of *Aspergillus fumigatus* meningitis was reported in Sri Lankan patients who received spinal anesthesia for a cesarean section. The examination of unused plastic syringes, needles, cannulas, and ampules of anesthetic agents confirmed that 43 syringes from three different manufactures were contaminated with *Aspergillus*. All of these supplies had been stored in a poorly maintained, humid warehouse. Although the exact source of the contamination remained unclear, inadequate storage facilities, owing to the mass influx of donations, was identified as the most plausible explanation [5]. Another investigation conducted in New Orleans after the Hurricanes Katrina and Rita in 2005 revealed high concentrations of molds, endotoxins, and fungal glucans in the environment. *Penicillium* and *Aspergillus* were the most commonly fungal genera cultured from the indoor air system following these natural events [6]. It is worth mentioning that our patient presented with aspergillosis in September 2018, right after Hurricane Irma in Miami, Florida. Our hospital was not affected by flooding and we did not identify any other cases of post-surgical aspergillosis. Despite having a high number of immunocompromised patients in our facility, we did not see any significant increase in the cases of pulmonary aspergillosis during that period. Additionally, no new building or renovation took place while the patient was hospitalized. However, our patient reported some damages to her house caused by the hurricane, which could have potentially increased the concentration of conidia in the air system. Unfortunately, no investigations were performed on the patient’s home and the contribution of this natural disaster to the development of aspergillosis remains unknown.

One of the factors that is classically associated with aspergillosis is immunosuppression, however cases of post-surgical aspergillosis are mostly described in immunocompetent individuals. According to the literature, 52% of patients with aspergillosis post-neurosurgery report the use of corticosteroids prior to surgery [1]. This could possibly facilitate the propagation of this fungal infection. Our patient was started on steroids right after the surgery, which could have been a contributing factor to the development of aspergillosis. Although the ultimate source of aspergillosis in our patient could not be established, we speculate that probably high conidial inhalation at the patient’s home, with hematogenous seeding from the respiratory tract, may have led to involvement of the surgical site.

The diagnosis of post-surgical central nervous system (CNS) aspergillosis is very challenging. The affected patients can have different clinical presentations including meningitis, central nervous system abscesses, mycotic aneurysms, and cerebral infarction. Most of the patients have a subacute or chronic form of meningitis that goes unrecognized for several weeks and sometimes display a relapsing character [7]. Cases of acute meningitis with rapid deterioration have been described in patients that had direct inoculation of the fungus into the cerebrospinal fluid or subarachnoid space, such as the ones reported after spinal anesthesia [7]. In our patient, the clinic course was subacute, presenting 3 weeks post-surgery with nonspecific symptoms (e.g., fever and occasional headaches) and the development of florid manifestations of meningoencephalitis on the 4th day of her hospital stay. Given the nonspecific presentation of this condition, the diagnosis is established post-mortem in up to 32% of affected patients [1]. Non-culture based diagnostic methods are rarely helpful in this setting. CSF analysis shows pleocytosis in 95.3% of cases and neutrophilic predominance in 68.4% of cases. Hypoglycorrhachia and hyperproteinorrhachia are present in 62.5% and 50% of cases, respectively [7]. Culture from CSF has shown poor sensitivity. A literature review conducted by Antinori et al. found a sensitivity of 31% [7]. Some of the measures that appear to increase the culture yield are to obtain high volumes of CSF or to perform serial lumbar punctures [8,9]. In our patient, CSF culture did not show any growth, despite the numerous samples sent for this purpose.

Another diagnostic test that has shown good results is galactomannan from CSF. This test has been detected in CSF from patients with CNS aspergillosis caused by *A. fumigatus*, *Aspergillus terreus*, and *Aspergillus flavus* [10]. The sensitivity of this study can be as high as 87% and the specificity is nearly 100% [7,10]. However, false positive results have been reported in cases of *Fusarium*, *Penicillium*, and *Histoplasma*. In the review of 92 cases of *Aspergillus* meningitis conducted by Antinori et al., the median CSF galactomannan index was 6.58 (range 2.2–578), which is higher than what is typically observed in serum [7]. Some reports have shown that CSF galactomannan may also lead to an earlier diagnosis. Cases of galactomannan that have been detected in CSF 45 days and 9 months before positive cultures are well documented in the literature [11,12]. In addition to its diagnostic value, CSF galactomannan appears to be useful in evaluating treatment response. A decline in CSF titers have been observed after antifungal therapy, which correlates with good clinical outcomes [13]. Unfortunately, our microbiology laboratory was not able to perform galactomannan in CSF samples.

In recent years, molecular techniques have emerged as effective diagnostic tools for CNS aspergillosis. Many authors have reported excellent detection rates with polymerase chain reaction (PCR). Reinwald et al. evaluated the performance of an *Aspergillus*-specific nested PCR in CSF in eight patients with CNS aspergillosis, and found a detection rate of 100% [14]. Kami et al. have also reported outstanding results in a cohort of five patients that were tested with this technique [15]. When applied to other specimens such as pulmonary tissue, the sensitivity can be as high as 90% [14]. Despite these promising results, this molecular technique has not been standardized for widespread use. In our patient, *Aspergillus* was detected only by brain tissue PCR. Of note, fungal cultures from CSF and brain tissue did not show any growth, which demonstrates the high sensitivity of this test.

In the majority of cases, the diagnosis of CNS aspergillosis is achieved by histopathology, which typically shows septated hyaline hyphae with acute angle branching. Several molds, including *Scedosporium* spp and *Fusarium* spp, can have similar appearances in histopathologic sections. Since the treatment of infections caused by these fungi may differ, it is important to confirm genus and species by culture. Special histopathology techniques such as immunohistochemistry using an anti-*Aspergillus* antiserum, are highly specific for *Aspergillus* spp, however cross-reactivity with other molds has been reported. In our patient, the biopsy was able to identify septated molds, which prompted the initiation of antifungal therapy. However, ultimately the definitive diagnosis of aspergillosis was achieved by PCR.

The optimal treatment of post-surgical CNS aspergillosis involves aggressive clinical and surgical therapy. The crucial role of surgical intervention was evidenced by the study of Kourkoumpetis et al., in which the mortality rate decreased by 35% in patients who received surgical treatment when compared to individuals who were only medically treated [16]. Regarding antifungal therapy, voriconazole is the drug recommended by the Infectious Disease Society of America’s guideline. Of note, this recommendation is based primarily on open-labelled studies that are conducted on patients with hematologic disorders [17]. There is no specific mention in the guideline about the treatment of post-surgical cerebral aspergillosis. Another drug that has demonstrated favorable responses is amphotericin, and it remains as one of the preferred drugs in clinical practice, probably due to its broader antifungal coverage, which provides a clear advantage in cases of uncertain diagnosis [16,18]. Other reports have described the efficacy of posaconazole, itraconazole, and echinocandins. Some clinicians have used double antifungal therapy successfully, however there are no data suggesting better outcomes with this approach. The optimal duration of antifungal therapy remains unknown. Pasqualotto et al. have recommended a minimum of 3 months after the appropriate debridement of the infected tissue [1]. No prospective studies have been conducted to address this issue. A wide range have been reported in the literature, ranging from 8 weeks to 12 months of antifungal therapy [7].

The prognosis of post-surgical CNS aspergillosis is poor, with a mortality rate that can reach 68% [1]. These rates are even higher in immunocompromised individuals with disseminated aspergillosis affecting the CNS, in whom the mortality can be as high as 90% [1]. Withdrawing immunosuppressive medications and the aggressive surgical debridement are measures that are not usually feasible in patients with transplant or hematologic disorders, which may explain the worse outcomes observed in this population.

## 4. Conclusions

CNS aspergillosis is an uncommon complication following neurosurgical interventions that carries a high mortality rate. The nonspecific manifestations of this condition and its subacute or chronic onset usually leads to delayed identification and treatment. The diagnosis is very challenging and usually relies on histology and cultures. Molecular tests have emerged as promising diagnostic tools in recent years. Although *Aspergillus* PCR is a molecular technique that is not yet standardized, our case illustrates its usefulness and high sensitivity when applied to tissue specimens. The treatment of this condition involves the combination of surgical and medical measures. The optimal duration of antifungal therapy is unknown, however we have obtained good results with 3 months of voriconazole treatment.
